# Supplementary material for: Integrated proteomic and transcriptomic landscape of macrophages in mouse tissues
Source: Nat Commun. 2022 Nov 30;13:7389. doi: 10.1038/s41467-022-35095-7 (PMC9712610; doi:10.1038/s41467-022-35095-7)
Supplement: Supplementary file 1 — Supplementary Information [file 41467_2022_35095_MOESM1_ESM.pdf]

Supplementary Information File

Integrated proteomic and transcriptomic landscape of macrophages in mouse tissues

Jingbo Qie,[1, #] Yang Liu,[1, #] Yunzhi Wang,[1, #] Fan Zhang,[1, #] Zhaoyu Qin,[1] Sha Tian[1], Mingwei Liu,[2] Kai Li,[2] Wenhao Shi,[2] Lei Song,[2] Mingjun Sun,[1] Yexin Tong,[1] Ping Hu,[3] Tao Gong,[4] Qiong Xia,[4] Yi Huang,[4] Bolong Lin,[4] Xuesen Zheng,[4] Rongbin Zhou,[4] Jie Lv,[5] Changsheng Du,[5] Yi Wang,[2,6] Jun Qin,[1, 2, 6] Wenjun Yang,[3, *] Fuchu He,[1, 2, *] and Chen Ding[1, 2, *]

[1]State Key Laboratory of Genetic Engineering, Institutes of Biomedical Sciences, Human Phenome Institute, School of Life Sciences, Zhongshan Hospital, Fudan University, Shanghai 200433, China;

[2]State Key Laboratory of Proteomics, Beijing Proteome Research Center, National Center for Protein Sciences, Beijing 102206, China

[3]Department of Pediatric Orthopedics, Xin Hua Hospital Affiliated to Shanghai Jiao Tong University School of Medicine, Shanghai, 200092, China.

[4]Hefei National Laboratory for Physical Sciences at Microscale, the CAS Key Laboratory of Innate Immunity and Chronic Disease, School of Life Sciences, University of Science and Technology of China, Hefei 230027, China

[5]Putuo District People's Hospital, Shanghai Key Laboratory of Signaling and Disease Research, School of Life Sciences and Technology, Tongji University, Shanghai 200092, China

[6]Alkek Center for Molecular Discovery, Verna and Marrs McLean Department of Biochemistry and Molecular Biology, Department of Molecular and Cellular Biology, Baylor College of Medicine, Houston, Texas 77030

[#]These authors contributed equally

[*]Correspondence: chend@fudan.edu.cn (C.D.), hefc@nic.bmi.ac.cn (F.C.H.), wjyang@sibcb.ac.cn (W.J.Y.)

Supplementary Figure 1

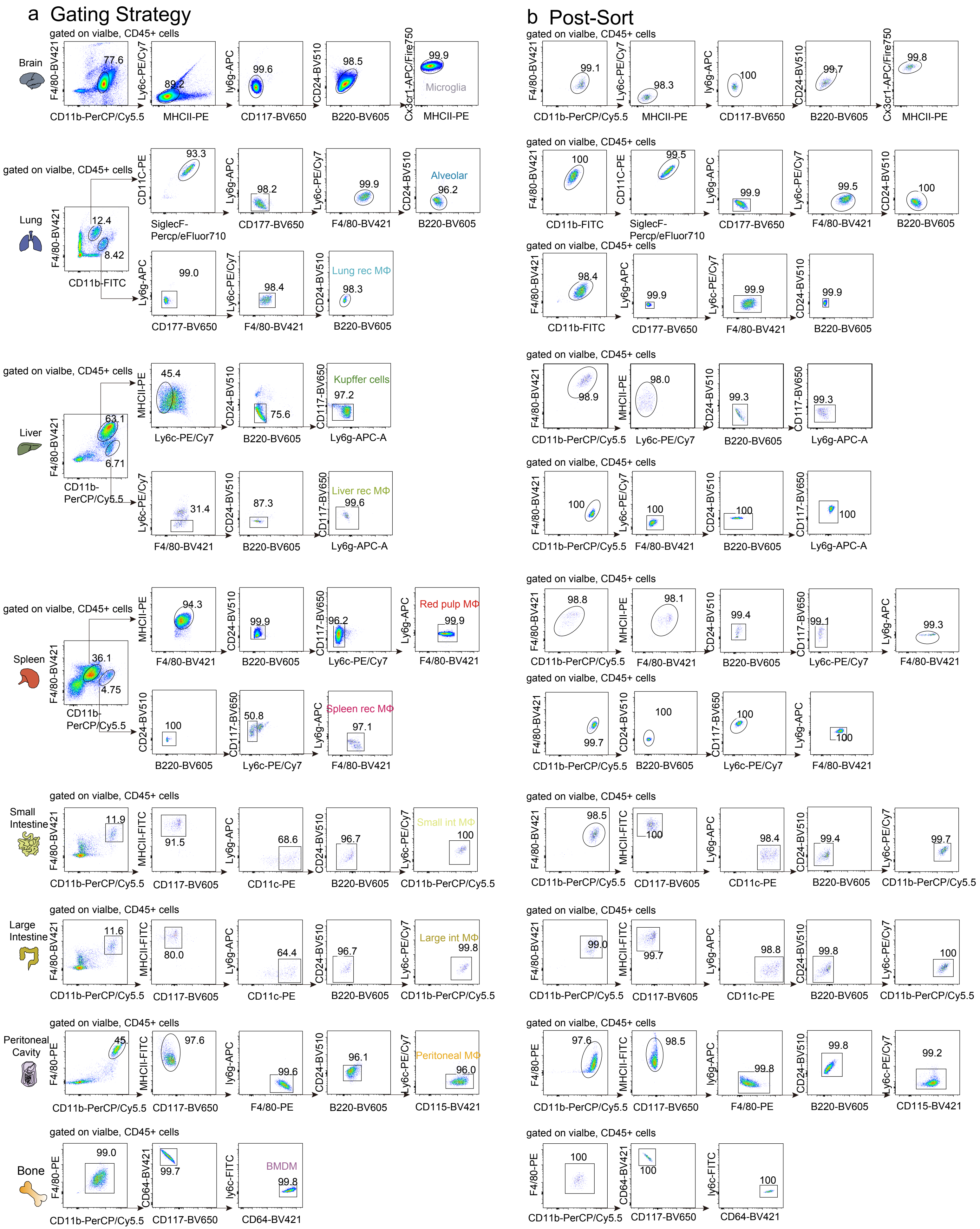

Supplementary Figure 1. Sorting strategies of 10 macrophage populations in 8 tissues, related to Figure 1. (**a**) flow cytometry (FCM) Sorting strategy for 10 primary macrophages and FCM test of BMDM, including microglia in the brain (dusty blue), alveolar macrophages and lung-recruited macrophages (blue), Kupffer cells and liver-recruited macrophages (green), spleen red pulp macrophages and spleen-recruited macrophages (red), small intestinal macrophages (light yellow), large intestinal macrophages (dark yellow), peritoneal macrophages (light pink) and BMDMs (orange). Cells were gated on viable, single-cell, and $CD45^+$ populations. (**b**) Representative post-sort FCM analysis of 10 primary macrophage populations and BMDM. Cells were gated on viable, single-cell, and $CD45^+$ populations.

Supplementary Figure 2

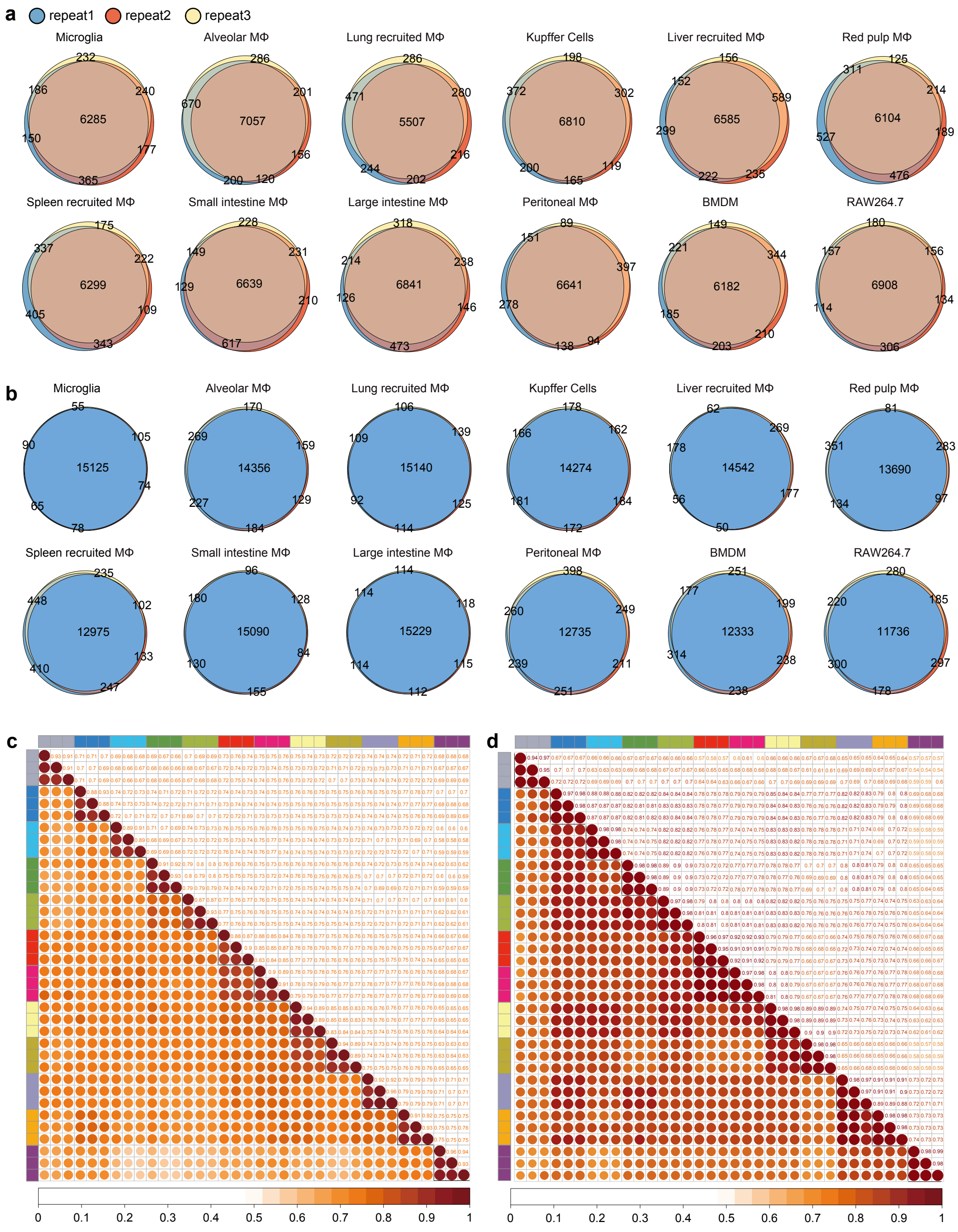

Supplementary Figure 2. Quality control of the macrophage proteome landscape, related to Figure 1. (**a, b**) Venn diagram of the numbers of identified genes in triplicated proteome (a) or transcriptome (b) dataset among 12 macrophage populations. (**c, d**) The matrix of Pearson correlation coefficients among triplicated proteome (c) or transcriptome (d) data of the12 macrophage populations. The color code follows the indicated values of correlation coefficients.

Supplementary Figure 3

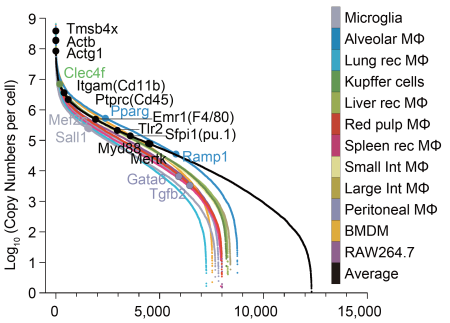

Supplementary Figure 3. Dynamic ranges of the proteomes of the 12 macrophage populations, based on the mean value of the estimated copy numbers. related to Figure 1.

Supplementary Figure 4

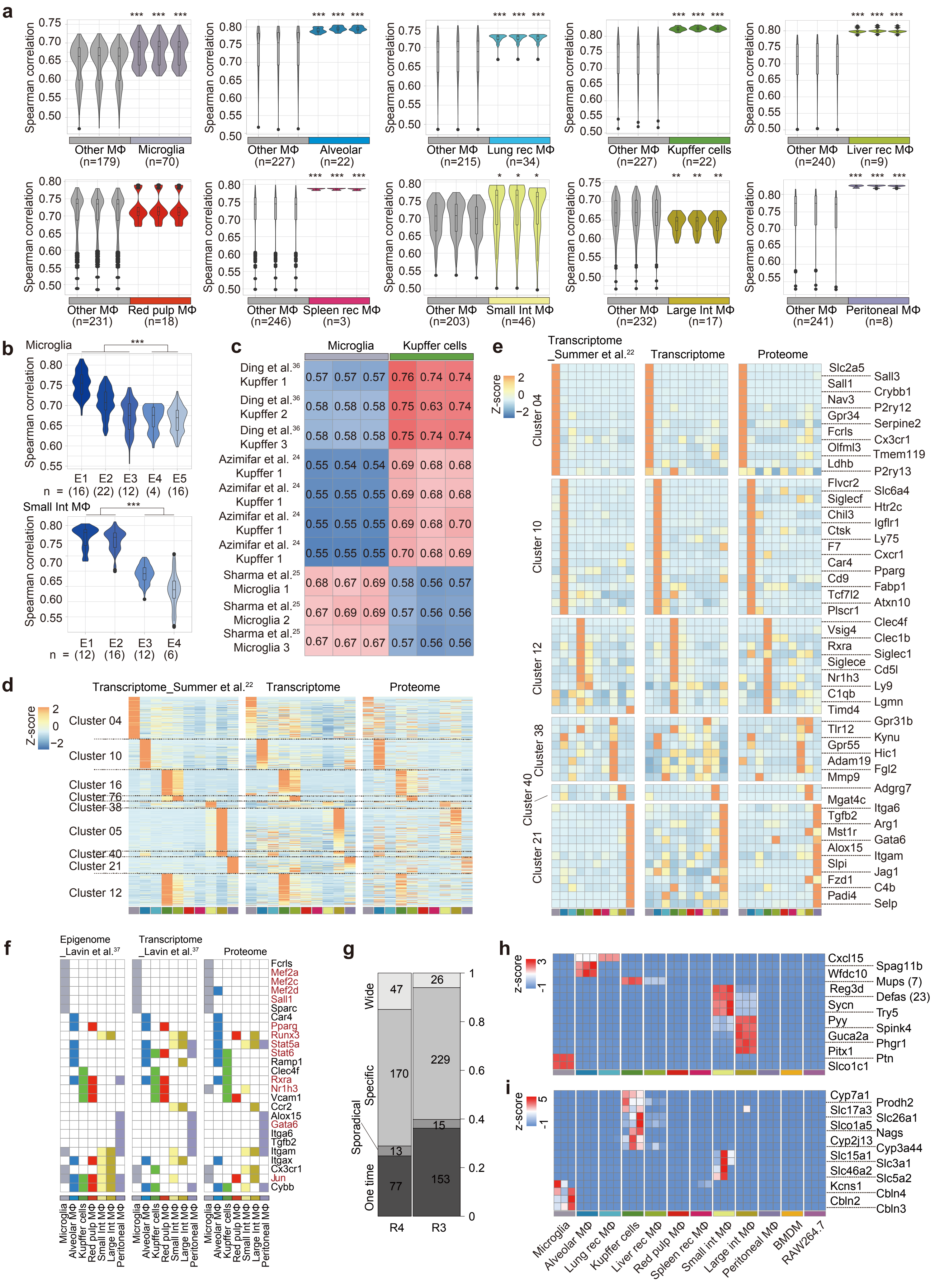

Supplementary Figure 4. Data comparison between published research and our proteome or transcriptome datasets, related to Figure 1. (a) Violin plots of Spearman correlation coefficient *r* values between the transcriptomes of the indicated macrophage populations in a published meta-analysis[22] (average expression values) and in this study. Grey columns show coefficients between the transcriptome of the indicated population in our study and the transcriptomes of other populations in the meta-analysis. Interquartile ranges (IQRs) as boxes, with the median as a black line and the whiskers extending up to the most extreme points within 1.5-fold IQR, the outliers are shown as individual points. The numbers of involving datasets (n) are indicated in the boxplot, respectively. *$p < 0.05$, ** $p < 0.01$, *** $p < 0.001$ (two-sided Student's t-test). (**b**) Violin plots depicting correlations between our transcriptome and different published datasets (in the metadata[22]) of microglia or small intestinal macrophage populations. The BioProject ID of the published datasets for the groups labelled E1 to E5 in the microglia diagrams are PRJNA529096, PRJNA421946&PRJNA422281, PRJNA507265, PRJNA529095, and PRJNA506249. The BioProject IDs of the published datasets for the groups labeled E1 to E4 in the small intestinal macrophage diagrams are PRJNA471340, PRJEB27719, PRJNA591465, and PRJNA325288. Interquartile ranges (IQRs) as boxes, with the median as a black line and the whiskers extending up to the most extreme points within 1.5-fold IQR, the outliers are shown as individual points. The numbers of involving datasets (n) are indicated in the boxplot, respectively. *$p < 0.05$, ** $p < 0.01$, *** $p < 0.001$ (two-sided Student's t-test). **(c)** Spearman correlation coefficient matrix of the proteome between our datasets (average expression values) and those reported in published studies[24, 25, 36]. **(d, e)** Heatmap of the expression profiles of indicative gene clusters (d) and representative signatures (e) defined by the meta-analysis[22] in the published datasets as well as in our transcriptomic and proteomic datasets. The mean values for each gene in all populations are color-coded based on the z-scored TPM (for the transcriptome) and copy numbers per cell (for the proteome). **(f)** Expression of molecular markers of each macrophage in published epigenomic and transcriptomic datasets[37] and our proteomic datasets. The molecules in red represent transcription factors. The color represents the specific expression of the protein in relevant macrophage populations (by dividing the protein copy number in a certain macrophage by the average copy number of the 12 macrophage populations, fold change > 1.5). (**g**) The spinogram of protein products of 730 missing RNAs in R3 or R4 in Figure 1f. The region marked with 'One time' represents proteins identified only once over 36 experiments, 'Sporadical' for proteins identified nonrepetitively in different populations, 'Specific' region for proteins stably identified in one

or two populations, ‘Wide’ region for proteins stably identified in more than two macrophage populations. (**h, i**) Heatmap of the expression values of representative transcripts (h) or proteins (i) located in R4 in Figure 1f. The expression values for each gene in all populations are color-coded based on the z-scored copy numbers per cell. **Source data are provided as a Source Data file.**

Supplementary Figure 5

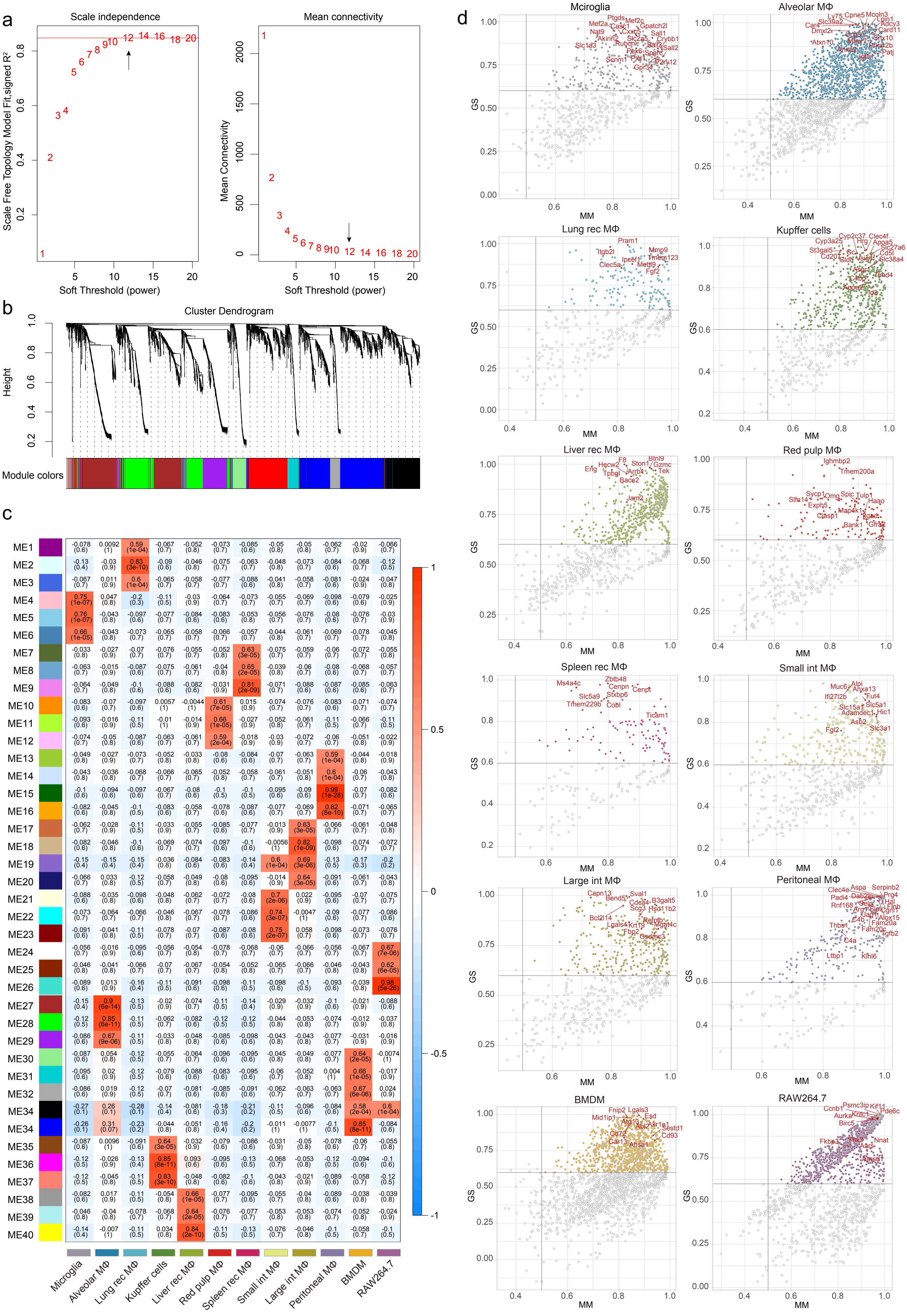

Supplementary Figure 5. weighted gene co-expression network analysis (WGCNA) of proteome patterns across the 12 macrophages. related to Figure 2. (**a**) Analysis of network topology for various soft-thresholding powers. The left panel shows the scale-free fit index (y-axis) as a function of the soft-thresholding power (x-axis). The right panel displays the mean connectivity (degree, y-axis) as a function of the soft-thresholding power (x-axis). A soft threshold at a power of 12 (based on scale-free topology model fit, $R^2 = 0.85$) was selected for module network construction. (**b**) Clustering dendrogram of genes, with dissimilarity based on the topological overlap, together with assigned module colors. (**c**) Module-macrophage associations. Each row corresponds to a module eigengene, column to a macrophage type. Each cell contains the corresponding correlation and *p*-value. The table is color-coded by correlation according to the color legend. (**d**) Scatterplots of Gene Significance (GS) for indicated macrophages vs. Module Membership (MM) in the indicated modules (with a correlation greater than 0.5) in Supplementary Figure 5c. Represent proteins with high GS or as gene signatures were labeled in red. **Source data are provided as a Source Data file.**

Supplementary Figure 6

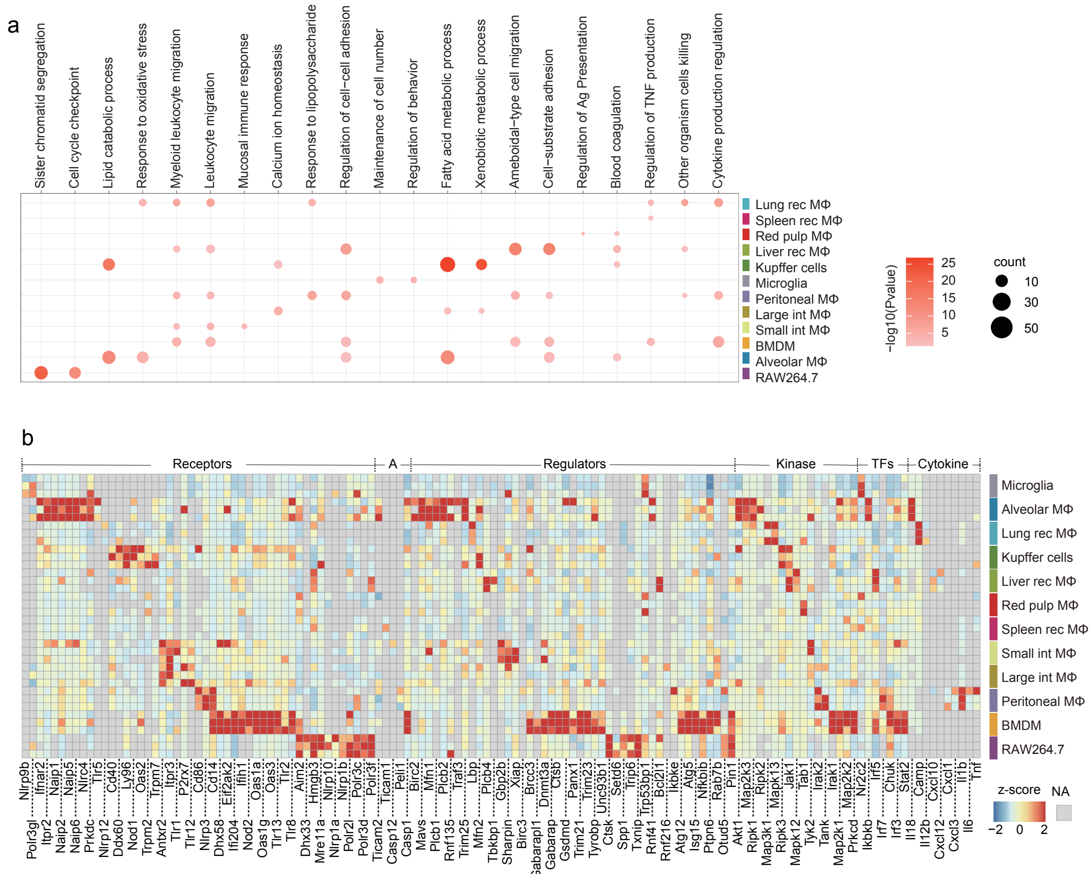

Supplementary Figure 6. Module function and expression pattern of PRR signaling pathway-related genes, related to Figure 2. (a) Representative functional annotations (GOBP and KEGG databases, One-sided Fisher's exact test, $p < 0.05$) for genes in the indicated CTMs in Figure 2b. The dot size represents the number of proteins involved in the relevant term. The color bar indicates the enrichment significance. **(b)** Heatmap of the expression patterns of PRR signaling pathway-related genes across the 12 macrophage populations. The expression values for each gene in all populations are color-coded based on the z-scored copy numbers per cell. The grey blocks represent missing values. **Source data are provided as a Source Data file.**

Supplementary Figure 7

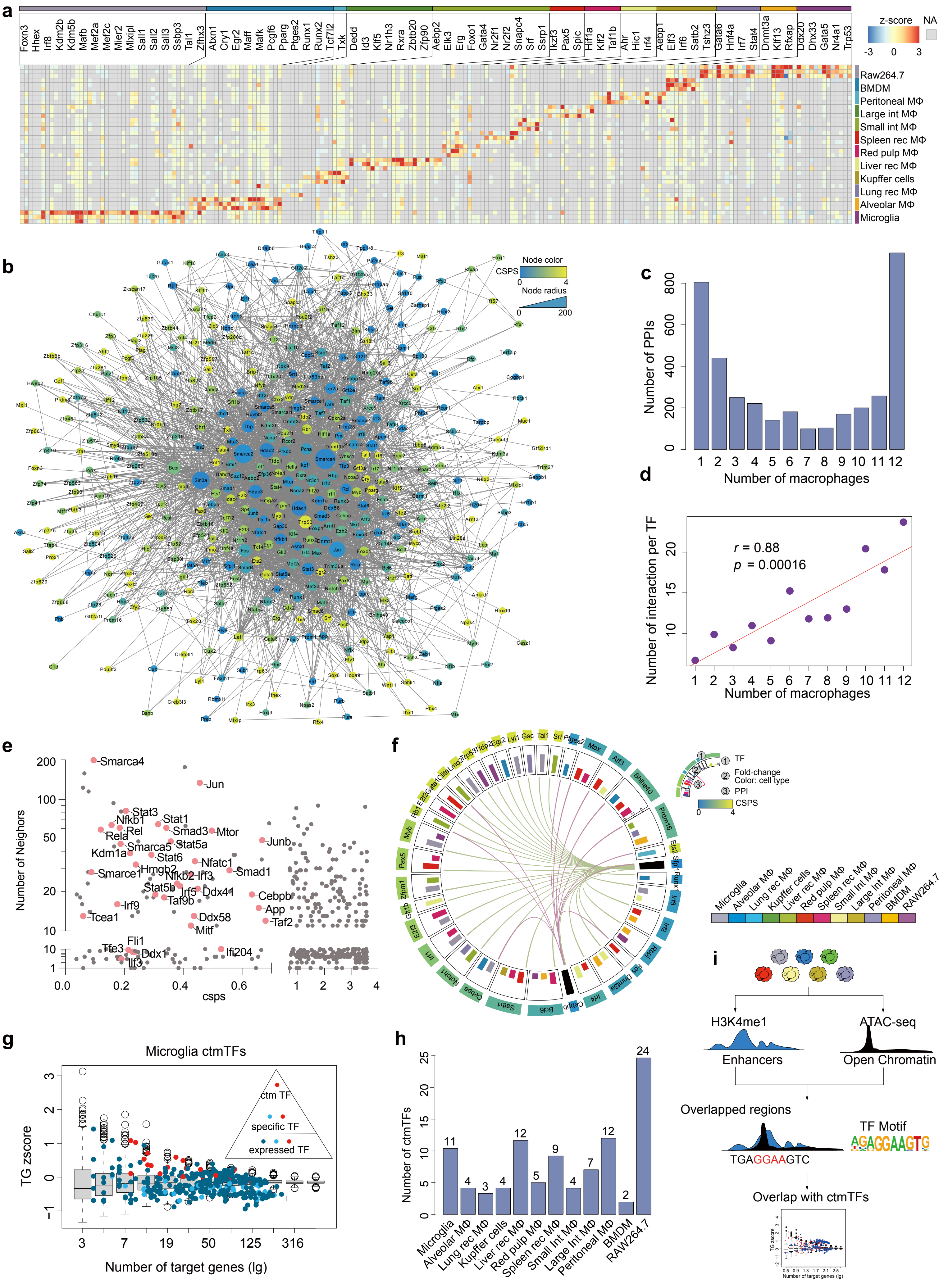

Supplementary Figure 7. Differential transcription factors (TFs) expression pattern in the 12 macrophage populations. Related to Figure 3. (a) Heatmap of specifically and ubiquitously expressed TFs in the 12 macrophage populations. The representative TFs are labeled at the top of each population. Expression values for each TFs at all analyzed samples are color-coded based on the intensities, low (blue) and high (red) z-scored copy numbers. The grey blocks represent missing values. (**b**) Network of TF-TF interactions of all TFs identified in the 12 macrophage populations. Node colors indicate the cell-specificity score (CSPS), and the node size shows the number of PPIs of the TF. (**c**) Diagram indicates the distribution of numbers of TF-TF interactions detected in the different number of macrophage populations. (**d**) The regression curve shows the correlation between the number of interactions per TF and the number of macrophages in which the TF was detected. The Pearson correlation coefficient is shown in the figure, $p = 0.00016$ (two-sided Pearson correlation test). (**e**) Ubiquitous TFs involved in the immune response in the GOBP database with CSPS $\leqslant$ 0.7. (**f**) The Interaction network between Cebpb, Sfpi1 (Pu.1) and macrophage-specific TFs. The outer colors indicate CSPS of the TF, the bars in the middle indicate the fold change of the TF's expression level in relevant populations versus the mean expression level in the 12 macrophage populations, the colors in the middle indicate the cell types and the inner lines indicate PPIs between TFs. (**g**) Boxplot coupled with scatter diagram showing the TF classification in microglia. Cell-type maintenance TFs (ctmTFs) are shown in red, cell-type-specific TFs are shown in light blue (except ctmTFs), and other TFs are shown in dark blue. Interquartile ranges (IQRs) as boxes, with the median as a black line and the whiskers extending up to the most extreme points within 1.5-fold IQR, the outliers are shown as individual points (n = 1000 digitally independent experiments). (**h**) Number of identified ctmTFs in each of 12 macrophage populations. (**i**) Schematic of the workflow to identify the key TFs in the combined analysis of proteome and epigenome. **Source data are provided as a Source Data file.**

Supplementary Figure 8

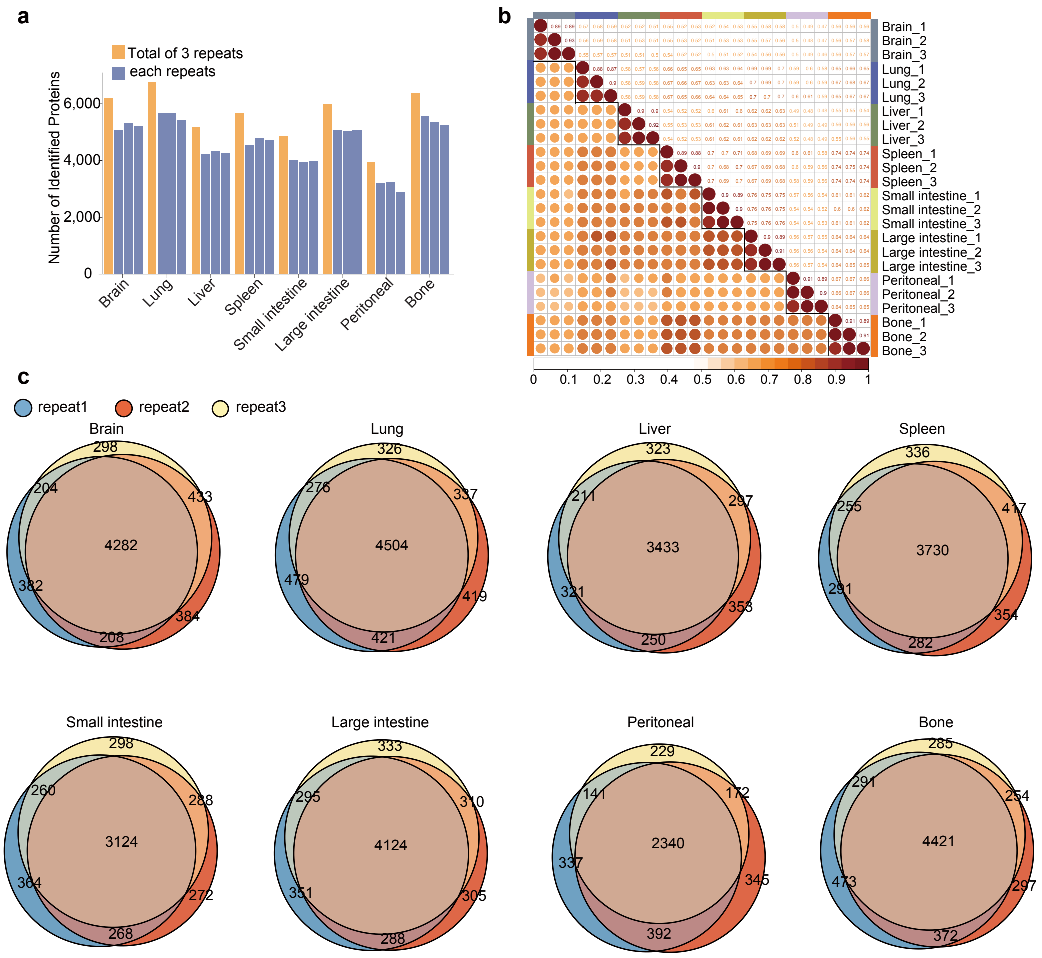

Supplementary Figure 8. Quality control of the Proteome profiling of eight tissues. Related to Figure 4. (**a**) Bar chart showing the number of identified proteins in the proteome data of 8 tissues. The blue bars represent the data for each replicate and the orange bars show the cumulative identification for each tissue. (**b**) The matrix of Pearson correlation coefficients among triplicated proteome data of 8 tissues. The color code follows the indicated values of the correlation coefficient. (**c**) Venn diagram of the numbers of identified proteins in triplicated proteome datasets among 8 tissues.

Supplementary Figure 9

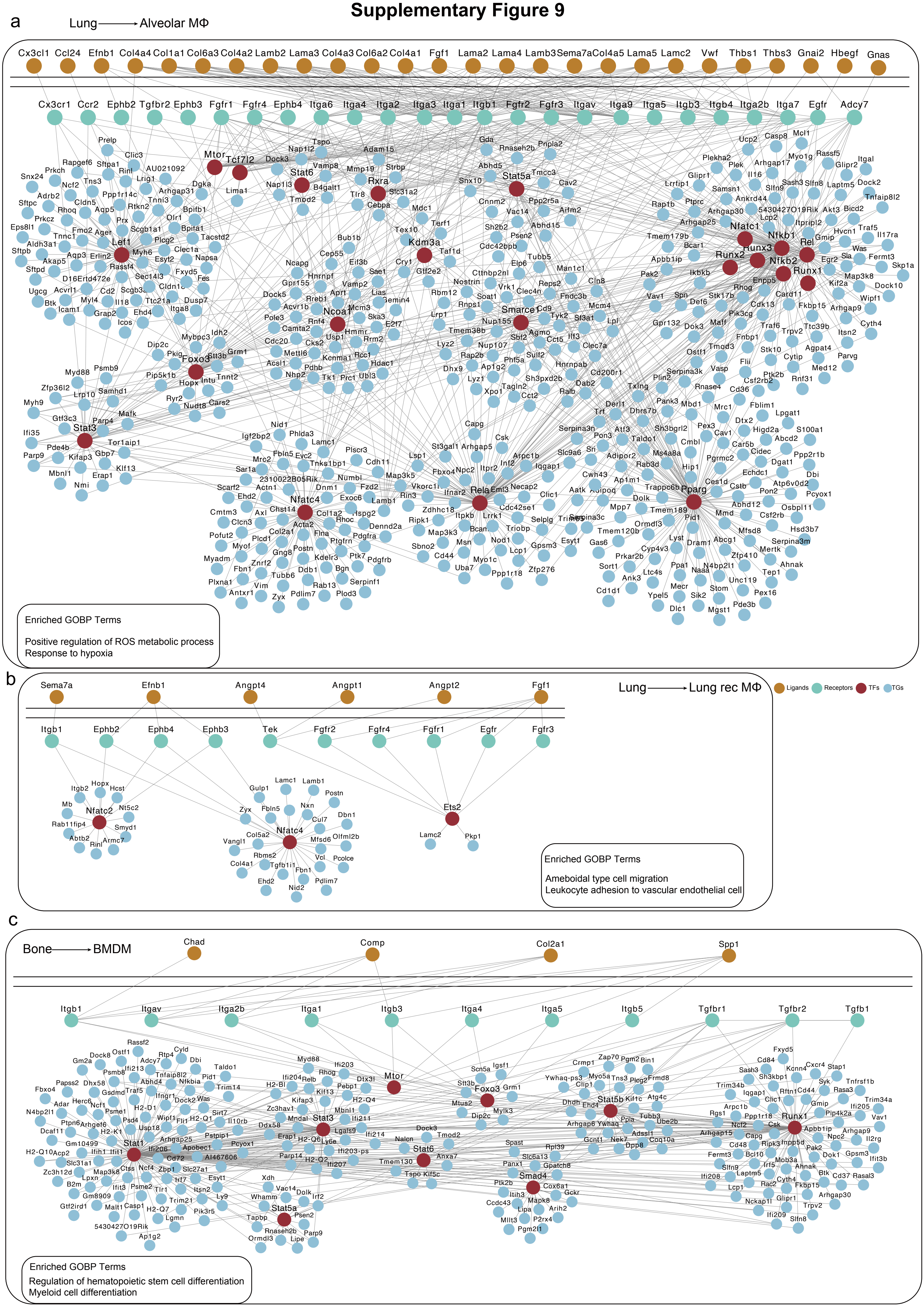

Supplementary Figure 10

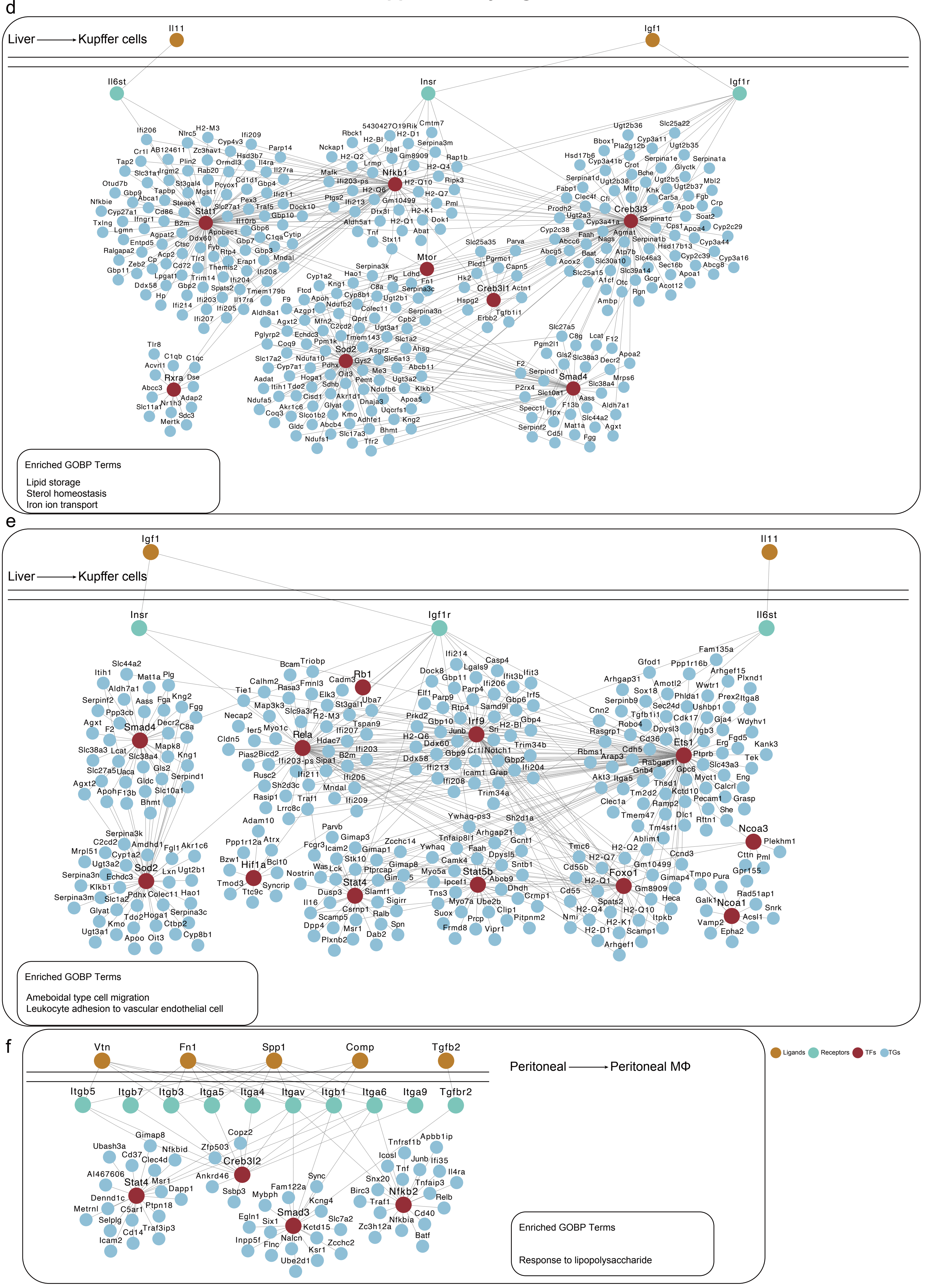

Supplementary Figure 11

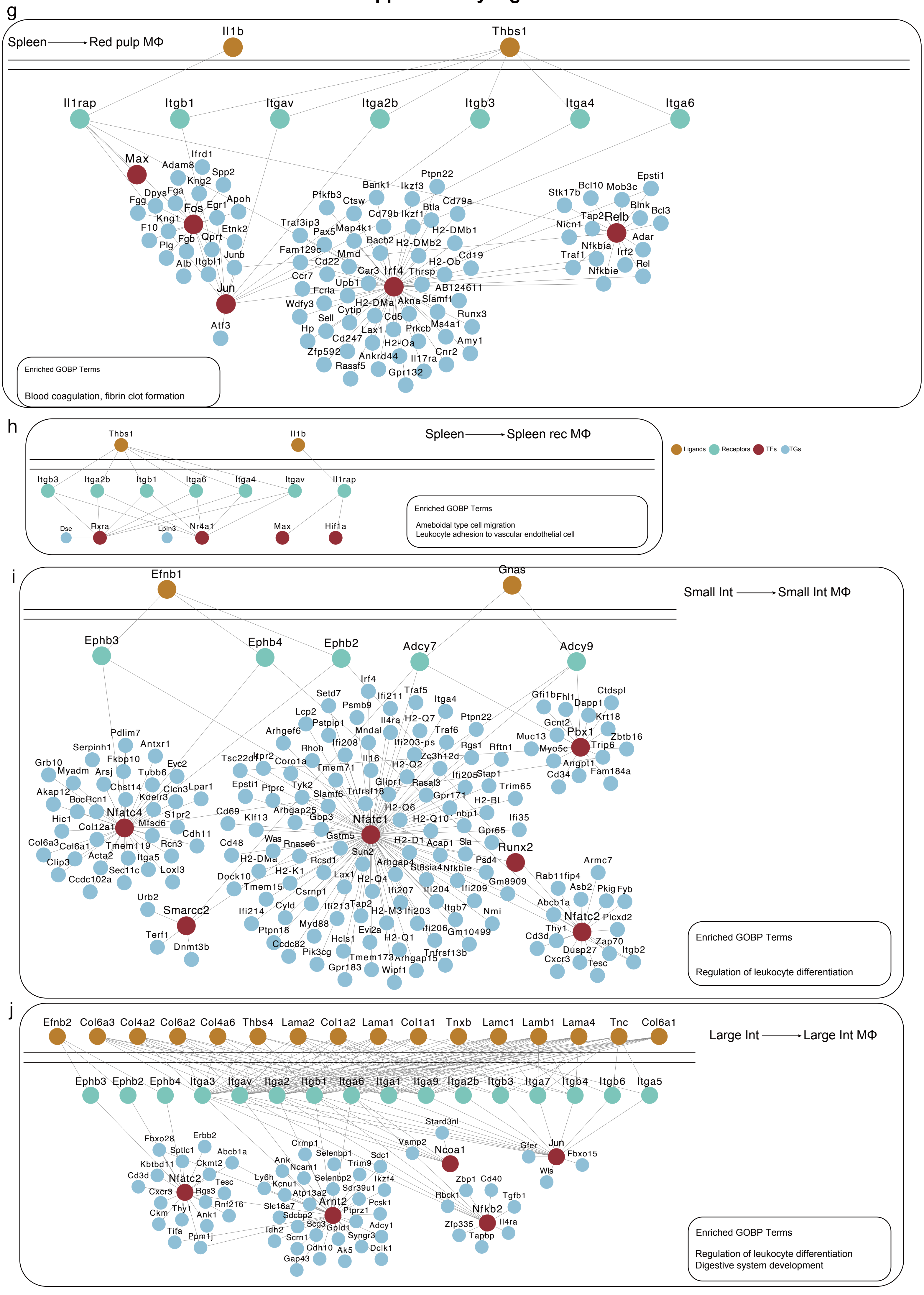

Supplementary Figure 9, 10, 11. Network of crosstalk between each macrophage population and the corresponding tissues. Related to Figure 4. (**a-j**) Network of crosstalk between the lung and alveolar macrophages (**a**), lung and lung-recruited macrophages (**b**), bone and BMDMs (**c**), Liver and Kupffer cells (**d**), Liver and liver-recruited macrophages (**e**), ascetic fluid and peritoneal macrophages (**f**), spleen and spleen red pulp macrophages (**g**), spleen and spleen-recruited macrophages (**h**), and intestine and intestinal macrophages (**i, j**). Orange indicates ligands, green indicates receptors, red indicates TFs, and blue indicates TGs. Proteins between receptors and TFs were ignored. Functions illustrated in Figure 3e for indicated macrophage populations are marked at the bottom.

Supplementary Figure 12

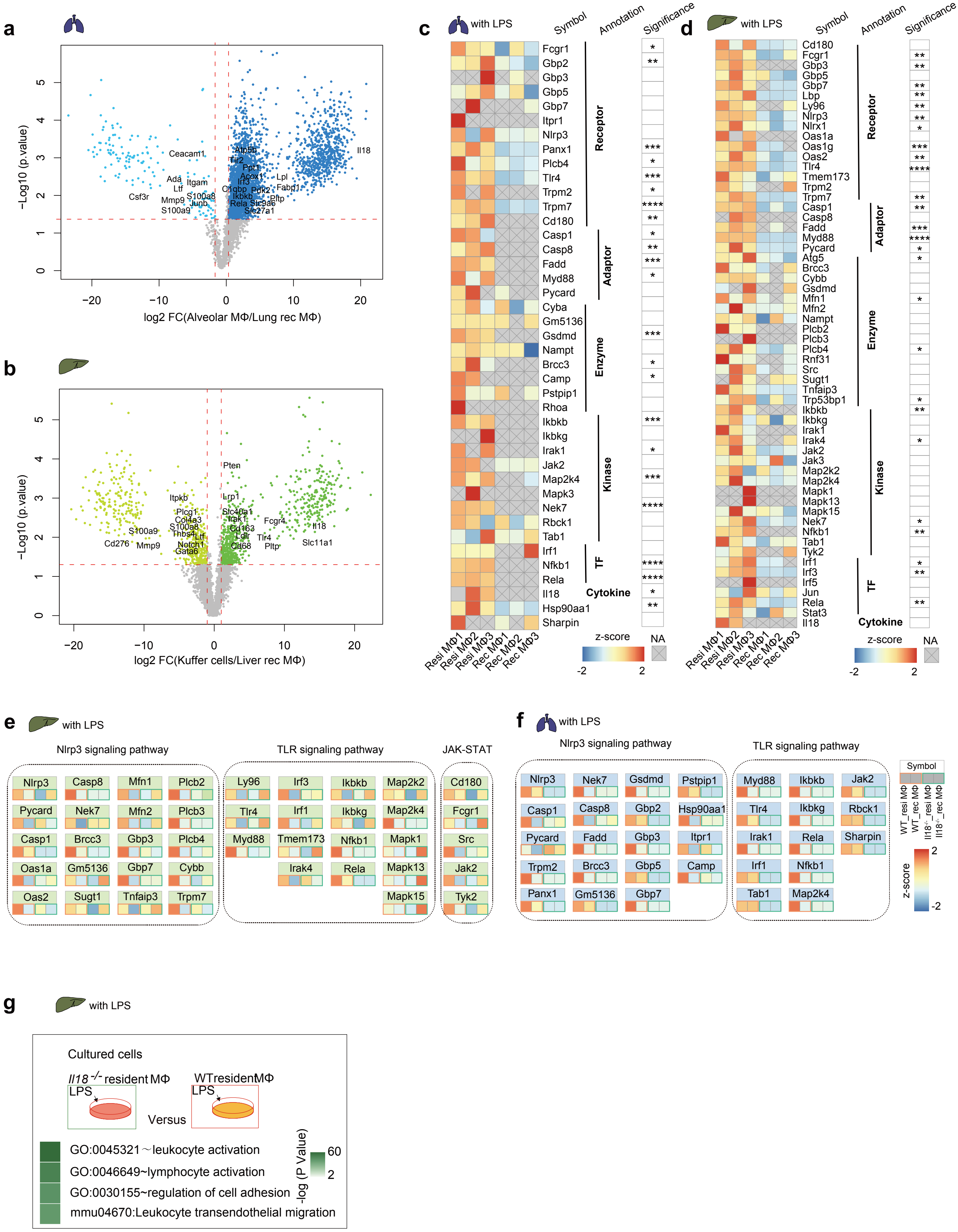

Supplementary Figure 12. Diverse molecular signatures between tissue-resident and recruited macrophages suggested by LC-MS. Related to Figure 7 and Figure 8. (**a, b**) Volcano plots of relative expression in tissue-resident and recruited macrophages with differential (> 2-fold), statistically significant ($p < 0.01$, two-sided Student's t-test) proteins in the lung (a) and the liver (b). **(c, d)** Heatmap showing the predominantly expressed proteins participating in inflammasome pathway (related to il18 secretion/production) in the tissue-resident macrophages compared to the tissue-recruited macrophages, in the lung (c) and liver (d), under LPS stimulation. Expression values for each protein in all analyzed samples are color-coded based on the intensities, low (blue) and high (red) z-score copy numbers. The grey blocks represent missing values. Protein categories and significance are labeled right. *$p < 0.1$, ** $p < 0.05$, *** $p < 0.01$, **** $p < 0.001$ (two-sided Student's t-test). (**e, f**) Gene panels indicate the differentially expressed proteins participating in the inflammasome pathway (related to il18 secretion/production) of the tissue-resident and recruited macrophages of wild-type and $Il18^{-/-}$ mice, in the liver (e) and lung (f), under LPS stimulation. Expression values for each protein in all analyzed samples are color-coded based on the intensities, low (blue) and high (red) z-score copy numbers. (**g**) The GO terms enriched by proteins highly expressed in the Kupffer cells of $Il18^{-/-}$ mice (versus wild-type one) under LPS stimulation (One-sided Fisher's exact test, $p < 0.05$). **Source data are provided as a Source Data file.**

Supplementary Figure 13

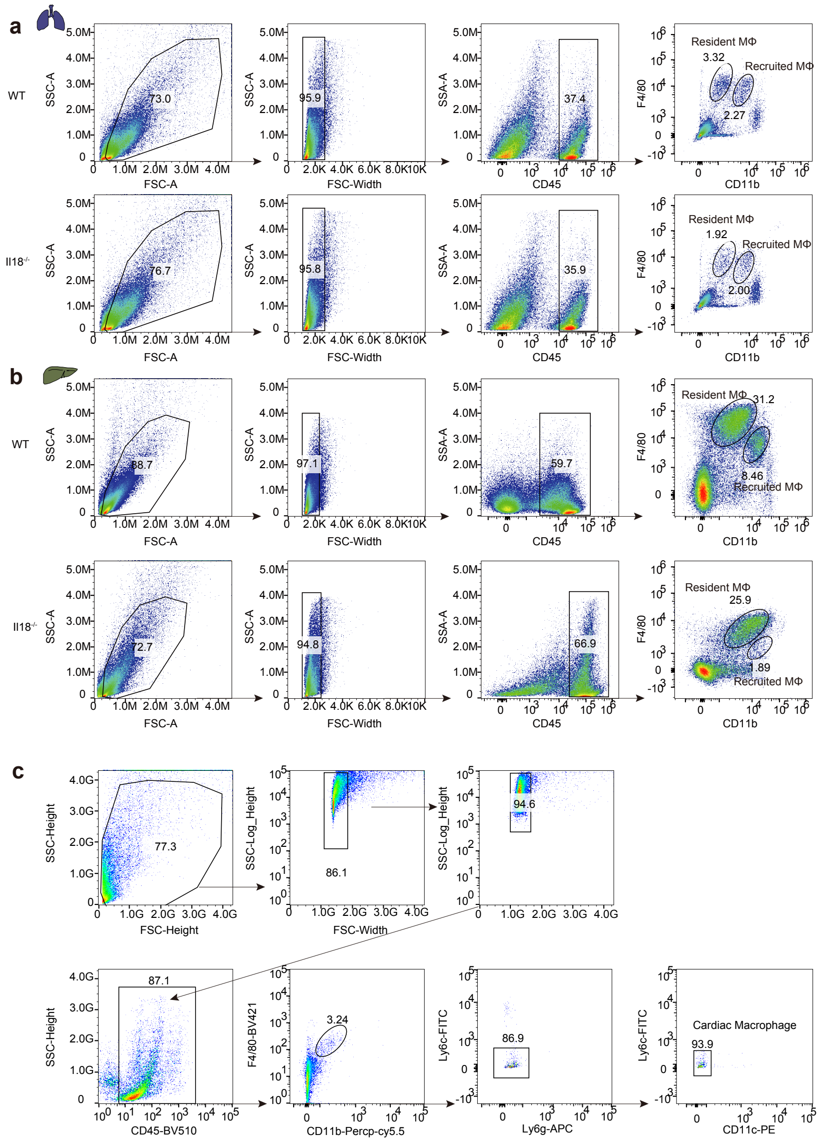

Supplementary Figure 13. Gating strategies of macrophages. (a, b) Gating strategies of macrophages in the lung (a) and liver (b), in the WT and *Il18*$^{-/-}$ mice. The same strategy was used for tissue-resident and recruited macrophage populations in Fig. 7a, 7i, and 8c. (c) Gating strategy of cardiac macrophage populations. Related to Figure 7 and Figure 8.

Supplementary Figure 14

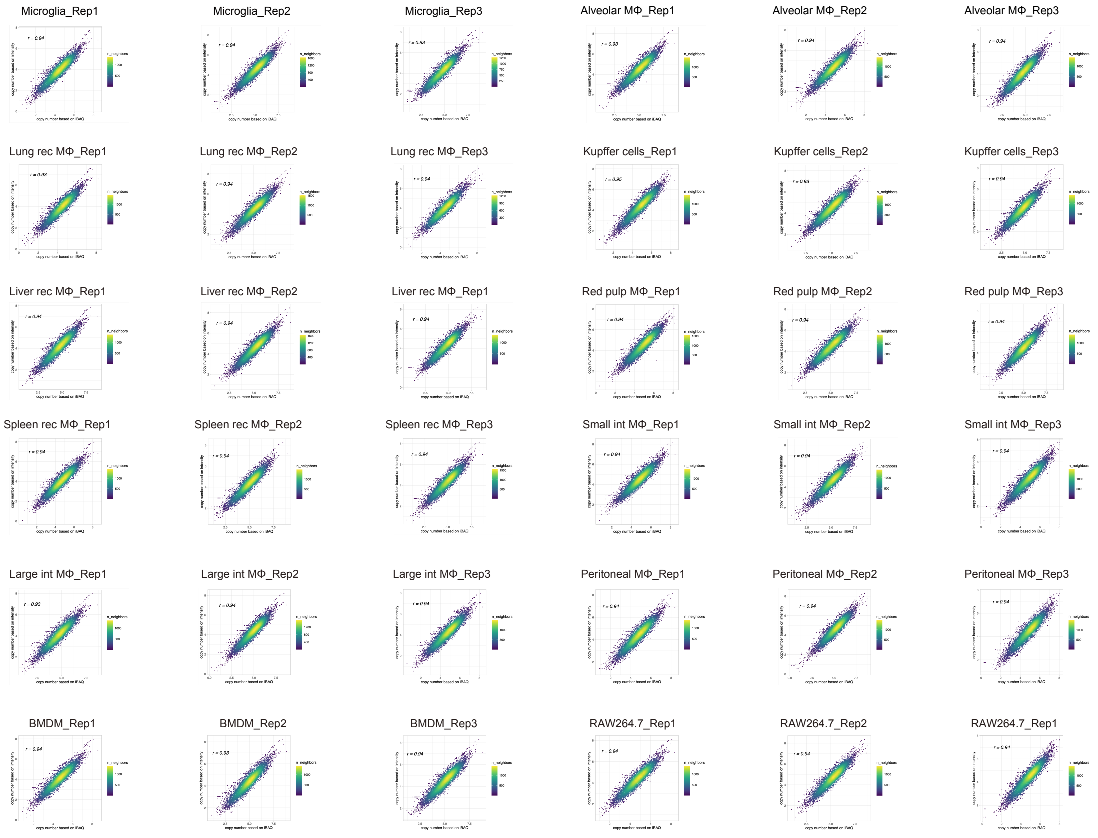

Supplementary Figure 14. The point charts show the Pearson correlation coefficients between the copy number values based on iBAQ (x-axis) and raw intensity (y-axis) in 36 proteome patterns for the 12 macrophage populations.

Supplementary Table 1. Antibodies used for cell sorting.

Name	Conjugate/Tag	Clone	Cat#	Company	Dilution
**B220**	Brilliant Violet 605	RA3-6B2	103243	Biolegend	1:200
**CD16/32 Block**	NA	2.4G2	553142	BD Biosciences	1:100
**CD115**	Brilliant Violet 421	AFS98	135513	Biolegend	1:200
**CD117**	Brilliant Violet 650	2B8	105853	Biolegend	1:300
**CD11b**	Percp-Cy5.5	M1/70	550993	BD Biosciences	1:200
**CD11b**	FITC	M1/70	557396	BD Biosciences	1:300
**CD11c**	PE	N418	12-0114-81	Invitrogen	1:200
**CD11c**	PE	N418	12-0114-82	Invitrogen	1:200
**CD24**	Brilliant Violet 510	M1/69	101831	Biolegend	1:200
**CD45**	CoraLite®488	30-F11	CL488-65087	proteintch	1:200
**CD45**	APC/Cyanine7	30-F11	103115	Biolegend	1:200
**CD45**	Brilliant Violet 510	30-F11	103137	Biolegend	1:200
**CD64**	Brilliant Violet 421	X54-5/7.1	139309	Biolegend	1:200
**Clec5a**	Alexa Fluor 488	226402	FAB1639G	R&D Systems	1:200
**Cx3cr1**	APC-Fire750	SA011F11	149039	Biolegend	1:200
**F4/80**	Brilliant Violet 421	BM8	123131	Biolegend	1:200
**F4/80**	PE	T45-2342	565410	BD Biosciences	1:200
**Ly6c**	PE-Cy7	HK1.4	25-5932-82	Invitrogen	1:200
**Ly6c**	FITC	HK1.4	128005	Biolegend	1:300
**Ly6g**	APC	1A8	560599	BD Biosciences	1:200
**Ly6g**	FITC	1A8	11-9668-82	Invitrogen	1:300
**Marco**	FITC	579511	FAB2956F	R&D Systems	1:200
**MHCII**	PE	M5/114.15.2	12-5321-82	Invitrogen	1:200
**MHCII**	FITC	M5/114.15.2	107605	Biolegend	1:200
**Muc1**	FITC	955	NBP2-47884F	Novus biologiclas	1:200
**Pdl1**	FITC	929903	FAB9078G	R&D Systems'	1:200
**Sifglec-F**	Percp-eFluor 710	1RNM44N	46-1702-82	Invitrogen	1:200

Supplementary Table 2. Gating panels and purity for all macrophages.

Tissue	cell type	Markers for identification/purification
**Brain**	Microglia	CD45+F4/80+CD11b+Cx3cr1hiMHCIIloLy6g-CD117-CD24-B220-Ly6clo
**Lung**	Lung-resident MΦ	CD45+F4/80hiCD11bloSiglecFhiCD11chiLy6g-CD117-CD24-B220-Ly6clo
	Lung-recruited MΦ	CD45+F4/80loCD11bhiLy6g-CD117-CD24-B220-Ly6chi/lo
**Liver**	Kupffer cells	CD45+F4/80hiCD11bloMHCII+Ly6g-CD117-CD24-B220-Ly6clo
	Liver-recruited MΦ	CD45+F4/80loCD11bhiLy6g-CD117-CD24-B220-Ly6chi/lo
**Spleen**	Spleen-resident MΦ	CD45+F4/80hiCD11bloMHCII+Ly6g-CD117-CD24-B220-Ly6clo
	Liver-recruited MΦ	CD45+F4/80loCD11bhiLy6g-CD117-CD24-B220-Ly6chi/lo
**SI**	Small intestinal MΦ	CD45+F4/80+CD11b+MHCII+CD11c+Ly6g-CD117-CD24-B220-Ly6clo
**LI**	Large intestinal MΦ	CD45+F4/80+CD11b+MHCII+CD11c+Ly6g-CD117-CD24-B220-Ly6clo
**Peritoneal**	Peritoneal MΦ	CD45+F4/80+CD11b+MHCIIloCD115+Ly6g-CD117-CD24-B220-Ly6clo
**Bone**	BMDMs	CD45+F4/80+CD11b+CD64+Ly6g-CD117-

Supplementary Table 3. Cell number and purity of post-sort macrophage populations in different tissues (per mouse).

Tissue	Number of MΦ/mouse	Purity
**Brain**	1e5-2e5	98%
**Lung**	4e5-5e5	98%
**Liver**	8e5-1e6	98%
**Spleen**	3e5-5e5	98%
**Small intestine**	3e5-3.5e5	98%
**Large intestine**	2.5e5-3e5	98%
**Peritoneal dropsy**	2e6-3e6	98%
