# Supplementary material for: Integrated proteomic and transcriptomic landscape of macrophages in mouse tissues
Source: Nat Commun. 2022 Nov 30;13:7389. doi: 10.1038/s41467-022-35095-7 (PMC9712610; doi:10.1038/s41467-022-35095-7)
Supplement: Supplementary file 3 — Description of Additional Supplementary Files [file 41467_2022_35095_MOESM3_ESM.pdf]

Description of Additional Supplementary Files

Supplementary Data 1: Proteome data of the 12 macrophage populations, the copy number values are shown for protein quantification. Related to Figure 1.

Column	Name	Description
1	Gene ID	Gene ID
2	Symbol	Gene symbol
3-38	Copy number	Copy number value of one protein in the indicated macrophage population/repeats.

Supplementary Data 2: Copy number value of transcriptome data of the 12 macrophage populations, genes with FPKM ⩾ 1 in at least one type of macrophage are included. Related to Figure 1.

Column	Name	Description
1	Gene ID	Gene ID
2	Symbol	Gene symbol
3-38	Copy number	Copy number value of one transcript in the indicated macrophage population/repeats.

Supplementary Data 3: Spearman correlation coefficients matrix between our transcriptome and published meta-data[22]. Related to Figure 1.

Column	Name	Description
1	Sample ID	Annotation of samples in meta-data
2	Cell type	Cell type samples in meta-data
3	experimentID	Annotation of research in meta-data clustered by different cell type and BioProjectID
4	Tissue/cell description	Samples names in published meta-data
5	SRA sample ID	Sample ID in GEO database
6	GEO accession	Dataset ID in GEO database

7	BioProject ID	Project ID in GEO database
8	Breed	Genetic background of mouse
9	Other information	Annotation of samples by the published meta-data
10	Tissue source	Tissue source
11-40	Correlation	Spearman correlation coefficients between indicated samples

Supplementary Data 4: Functional co-expressed modules of proteins identified in the proteome pattern of the 12 macrophage populations. Related to Figure 2.

Column	Name	Description
1	Symbol	Gene symbol
2	Celltype	Cell type of macrophage in which the protein is predominantly expressed.
3	moduleColor	Module colors of the WGCNA as shown in Supplementary Figure 5c
4	moduleNumber	Module numbers of the WGCNA
5	GS	Gene significance of the gene assigned to the indicated cell type in column 2
6	p.GS	*p* value of GS.
7	MM	Module membership
8	p.MM	*p* value of MM

Supplementary Data 5: Detailed information of TFs and ctmTFs identified in proteome patterns of each macrophage population. Related to Figure 3.

Column	Name	Description
1	Symbol	Gene symbol of the TFs
2	csps	cell-specificity score of the TF
3	ub. nub	Ubiquitous and non-ubiquitous TFs mark
4	cell-type specific TFs	macrophage type in which the TFs was identified as specific TFs.

5	ctmTF	macrophage type in which the TFs was identified as ctmTFs.
6	TG	Target genes of the TF identified in proteome datasets.

Supplementary Data 6: TF-TF interaction network. Related to Figure 3.

Supplementary Data 7: Proteome dataset of the eight tissues, including the brain, lung, liver, spleen, small intestine, large intestine, ascetic fluid, and bone marrow. The FOT values are shown for protein quantification. Related to Figure 4.

Column	Name	Description
1	Symbol	Gene symbol
2-37	Copy number	Copy number value of one protein in the indicated tissues/repeats.

Supplementary Data 8: Detailed information of networks of tissue-macrophage crosstalk in triplicate (in 12 sheets titled with macrophage type). Related to Figure 4.

Column	Name	Description
1	Symbol	Gene symbol of the source node
2	Symbol	Gene symbol of the target node
3	Type	Interaction type marked with LR: ligand to receptor, RT: receptor to TF, TT: TF to target gene
4	Pathway	Name KEGG pathway in which the edge involved
5	Repeats	Number of repeats

Supplementary Data 9: Proteome data of tissue-resident and recruited macrophages in the liver (Sheet 1) and lung (Sheet 2) of the wild-type mice under LPS stimulation *in vivo*. Related to Figure 7.

Column	Name	Description
1	Symbol	Gene symbol
2-7	Copy number	Copy number value of one protein in the indicated macrophage

		population/repeats.

Supplementary Data 10: Proteome data of tissue-resident and recruited macrophages in the liver (Sheet 1) and lung (Sheet 2) of the *Il18*$^{-/-}$ mice under LPS stimulation *in vivo*. Related to Figure 7.

Column	Name	Description
1	Symbol	Gene symbol
2-7	Copy number	Copy number value of one protein in the indicated macrophage population/repeats.

Supplementary Data 11: Proteome data of Kupffer cells in wild-type or *Il18*$^{-/-}$ mice under LPS stimulation *in vitro*. Related to Figure 8.

Column	Name	Description
1	Symbol	Gene symbol
2-13	Copy number	Copy number value of one protein in the indicated macrophage population/repeats.
