# Supplementary material for: Integrated proteomic and transcriptomic landscape of macrophages in mouse tissues
Source: Nat Commun. 2022 Nov 30;13:7389. doi: 10.1038/s41467-022-35095-7 (PMC9712610; doi:10.1038/s41467-022-35095-7)
Supplement: Supplementary file 15 — Reporting Summary [file 41467_2022_35095_MOESM15_ESM.pdf]

nature portfolio

Corresponding author(s): Chen Ding

Last updated by author(s): Nov 6, 2022

Reporting Summary

Nature Portfolio wishes to improve the reproducibility of the work that we publish. This form provides structure for consistency and transparency in reporting. For further information on Nature Portfolio policies, see our Editorial Policies and the Editorial Policy Checklist.

Statistics

For all statistical analyses, confirm that the following items are present in the figure legend, table legend, main text, or Methods section.

n/a	Confirmed	
☐	☒	The exact sample size (*n*) for each experimental group/condition, given as a discrete number and unit of measurement
☐	☒	A statement on whether measurements were taken from distinct samples or whether the same sample was measured repeatedly
☐	☒	The statistical test(s) used AND whether they are one- or two-sided<br>*Only common tests should be described solely by name; describe more complex techniques in the Methods section.*
☐	☒	A description of all covariates tested
☐	☒	A description of any assumptions or corrections, such as tests of normality and adjustment for multiple comparisons
☐	☒	A full description of the statistical parameters including central tendency (e.g. means) or other basic estimates (e.g. regression coefficient) AND variation (e.g. standard deviation) or associated estimates of uncertainty (e.g. confidence intervals)
☐	☒	For null hypothesis testing, the test statistic (e.g. F, t, r) with confidence intervals, effect sizes, degrees of freedom and P value noted<br>*Give P values as exact values whenever suitable.*
☒	☐	For Bayesian analysis, information on the choice of priors and Markov chain Monte Carlo settings
☐	☒	For hierarchical and complex designs, identification of the appropriate level for tests and full reporting of outcomes
☐	☒	Estimates of effect sizes (e.g. Cohen's *d*, Pearson's *r*), indicating how they were calculated

Our web collection on statistics for biologists contains articles on many of the points above.

Software and code

Policy information about availability of computer code

Data collection	The proteome data were performed by orbitrap fusion or orbitrap fusion lumos , Thermo fisher, with the software Xcalibur 4.2 (Thermo Fischer Scientific). The transcriptome data were obtained through MGISEQ2000 platform (The Beijing Genomics Institute, BGI).
Data analysis	database searching and protein quantification for the mass spectrometry raw data were performed using Firmiana proteomics workstation; transcripts quantification were performed using RSEM version 1.3.3. Statistical analyses were realized by R (v4.0.0). Approaches or algorithms used for the gene annotation include GSEA R package (v1.34.0), clusterProfiler R package (v3.14.3). Unsupervised clustering was performed using R package pheatmap (version 1.0.12). Proteins were clustered into functional modules using a weighted gene co-expression network analysis (WGCNA), with the R package WGCNA (1.71). PCA was performed to visualize separation of different macrophage populations in the statistical analysis environment R version 4.0.0 (with the function prcomp). We used the STRING database (https://cn.string-db.org) to explore the protein-protein interactions between TFs in 12 macrophages. The method named CCCEXPLOR (PMID: 25704820) was used to derive the hierarchical crosstalk network between macrophages populations and relevant tissue. Results from flow cytometric analysis were acquired using Beckman Counter cytoFLEX LX, or sorted by BD influx or BD FACS AriaIII with 100-μm nozzle. flow cytometry (FCM) data were analyzed by FlowJoTM 10.7.1. Standard statistical tests were used to analyze the data, including but not limited to Student's t test, Wilcoxon rank-sum test, Fisher's exact test, Pearson's correlation test, Spearman's rank correlation test. Unless otherwise specified, all statistical tests were two-sided. To account for multiple-testing, the p values were adjusted using the Benjamini–Hochberg FDR correction. All the Statistical analyses of clinical data were performed in R and GraphPad Prism. For functional experiments, each was repeated at least three times independently, and results were expressed as mean ± SD.

For manuscripts utilizing custom algorithms or software that are central to the research but not yet described in published literature, software must be made available to editors and reviewers. We strongly encourage code deposition in a community repository (e.g. GitHub). See the Nature Portfolio guidelines for submitting code & software for further information.

Data

Policy information about availability of data

All manuscripts must include a data availability statement. This statement should provide the following information, where applicable:

- Accession codes, unique identifiers, or web links for publicly available datasets
- A description of any restrictions on data availability
- For clinical datasets or third party data, please ensure that the statement adheres to our policy

All data generated in this study, including the raw files and quantitative data matrix of proteomes and transcriptomes, have been deposited online. Specifically, proteome datasets of the 12 macrophage populations and the proteome profiles of the eight tissue/organs have been deposited to iProX with accession number IPX0001245000 (https://www.iprox.cn), and can be archived through accession number PXD021583 in PRIDE database (https://www.ebi.ac.uk/pride). Proteome datasets of the macrophages in the liver and lung in wild-type or Il18-/- mice (related to Fig. 7 and Fig. 8) have been deposited to PRIDE with accession number PXD021657 (https://www.ebi.ac.uk/pride) . The RNA-seq data of the 12 macrophage populations are accessible in SRA with accession number PRJNA482293 (https://www.ncbi.nlm.nih.gov/sra).

The bulk RNA-seq datasets for data comparison between published and our datasets were derived from meta-analysis research published by Summers et al (PMID: 33031383). Raw ChIP-seq and ATAC-seq data were downloaded from GEO database (https://www.ncbi.nlm.nih.gov/geo) with the GEO accession number of GSE63339 and GSE63338, respectively, according to one study describing the enhancer landscape of the seven tissue-resident macrophage populations (PMID: 25480296). The ligand-receptor interactions were downloaded from the DLRP62 (http://www.hprd.org) and IUPHAR63 (http://www.guidetopharmacology.org) databases. We used the STRING database (https://cn.string-db.org) to explore the protein-protein interactions between TFs in 12 macrophages. The TF-TG regulatory network/relationship was archived from CellNET database (http://cellnet.hms.harvard.edu). Gene annotation analysis were performed based on Gene Ontology (http://geneontology.org) or KEGG database (https://www.genome.jp/kegg/) with the R Bioconductor package ‘clusterProfiler’ (R package v3.14.3).

Human research participants

Policy information about studies involving human research participants and Sex and Gender in Research.

Reporting on sex and gender	no human research participants
Population characteristics	no human research participants
Recruitment	no human research participants
Ethics oversight	no human research participants

Note that full information on the approval of the study protocol must also be provided in the manuscript.

Field-specific reporting

Please select the one below that is the best fit for your research. If you are not sure, read the appropriate sections before making your selection.

☒ Life sciences ☐ Behavioural & social sciences ☐ Ecological, evolutionary & environmental sciences

For a reference copy of the document with all sections, see nature.com/documents/nr-reporting-summary-flat.pdf

Life sciences study design

All studies must disclose on these points even when the disclosure is negative.

Sample size	We profiled the proteome patterns of 10 primary macrophage populations from 7 tissues, BMDM and the cell line RAW264.7. For each macrophage population, equal numbers of cells (1.5E6) were subjected to protein preparation for LC-MS/MS analysis.
Data exclusions	MS raw files were processed with the “Firmiana” (a one-stop proteomic cloud platform) against the mouse RefSeq protein database (updated on 04-07-2013) maintained by the National Center for Biotechnology Information (NCBI). The maximum number of missed cleavages was set to 2. Mass tolerances of 20 ppm for precursor and 0.5 Da for production were allowed. The fixed modification was cysteine carbamidomethylation and the variable modifications were N-acetylation and oxidation of methionine. For the quality control of proteins identification, the target-decoy-based strategy was applied to confirm that the FDR (False Discovery Rate) of both peptide and protein were lower than 1%. The program percolator was used to obtain the probability value (q-value), and showed that the FDR (measured by the decoy hits) of every peptide-spectrum match (PSM) was lower than 1%. Then all peptides shorter than seven amino acids were removed. The cutoff ion score for peptide identification was 20. All of the PSMs in all fractions were combined for protein quality control, which was a stringent quality control strategy. The q-values of both target and decoy peptide sequences were dynamically increased until the corresponding protein FDR was less than 1% employing the parsimony principle. Finally, to reduce the false positive rate, the proteins with at least one unique peptide were selected for further investigation.
Replication	We measured 3 biological replicates for each macrophage to obtain reliable proteome patterns. The proteome identifications of the 3

	replicates in each macrophage group obtained a high overlap and the average correlation coefficient of the biological replicates was Pearson R > 0.9.
Randomization	The mice with the same strain, age, sex and state for proteome processing were randomized and applied to cell isolation in the study.
Blinding	Not relevant with the project. No clinical data or samples included in this study.

Reporting for specific materials, systems and methods

We require information from authors about some types of materials, experimental systems and methods used in many studies. Here, indicate whether each material, system or method listed is relevant to your study. If you are not sure if a list item applies to your research, read the appropriate section before selecting a response.

Materials & experimental systems

n/a	Involved in the study
☐	☒ Antibodies
☐	☒ Eukaryotic cell lines
☒	☐ Palaeontology and archaeology
☐	☒ Animals and other organisms
☒	☐ Clinical data
☒	☐ Dual use research of concern

Methods

n/a	Involved in the study
☒	☐ ChIP-seq
☐	☒ Flow cytometry
☒	☐ MRI-based neuroimaging

Antibodies

Antibodies used

Name Conjugate/Tag Clone Cat# Company Dilution
B220 Brilliant Violet 605 RA3-6B2 103243 BD Biosciences 1:200
CD16/32 Block NA 2.4G2 553142 BD Biosciences 1:100
CD115 Brilliant Violet 421 AFS98 135513 Biolegend 1:200
CD117 Brilliant Violet 650 2B8 105853 Biolegend 1:300
CD11b Percp-Cy5.5 M1/70 550993 BD Biosciences 1:200
CD11b FITC M1/70 557396 BD Biosciences 1:300
CD11c PE N418 12-0114-81 Invitrogen 1:200
CD11c PE N418 12-0114-82 Invitrogen 1:200
CD24 Brilliant Violet 510 M1/69 101831 Biolegend 1:200
CD45 CoraLite®488 30-F11 CL488-65087 proteintch 1:200
CD45 APC/Cyanine7 30-F11 103115 Biolegend 1:200
CD45 Brilliant Violet 510 30-F11 103137 Biolegend 1:200
CD64 Brilliant Violet 421 X54-5/7.1 139309 Biolegend 1:200
Clec5a Alexa Fluor 488 226402 FAB1639G R&D Systems 1:200
Cx3cr1 APC-Fire750 SA011F11 149039 Biolegend 1:200
F4/80 Brilliant Violet 421 BM8 123131 Biolegend 1:200
F4/80 PE T45-2342 565410 BD Biosciences 1:200
Ly6c PE-Cy7 HK1.4 25-5932-82 Invitrogen 1:200
Ly6c FITC HK1.4 128005 Biolegend 1:300
Ly6g APC 1A8 560599 BD Biosciences 1:200
Ly6g FITC 1A8 11-9668-82 Invitrogen 1:300
Marco FITC 579511 FAB2956F R&D Systems 1:200
MHCII PE M5/114.15.2 12-5321-82 Invitrogen 1:200
MHCII FITC M5/114.15.2 107605 Biolegend 1:200
Muc1 FITC 955 NBP2-47884F Novus biologiclas 1:200
Pdl1 FITC 929903 FAB9078G R&D Systems' 1:200
Sifglec-F Percp-eFluor 710 1RNM44N 46-1702-82 Invitrogen 1:200

Goat anti-rabbit IgG-HRP (1:5000, abcam, catalog No: ab6721)or goat anti-mouse IgG-HRP (1:5000, abcam, catalog No: ab6789) were used as the secondary antibodies. Antibodies: Il1β (1:1000, CST, catalog No:12703), Il18 (1:1000, CST, catalog No:57058), Nek7 (1:1000, CST, catalog No:3057S), Nlrp3 (1:1000, CST, catalog No:15101), Pro-caspase (1:1000, CST, catalog No:2225S), β-actin (1:1000, CST, catalog No:3700S), anti-Il18 antibody (1:2000, BE0237, BioXCell).

Validation

1. B220-BV605 antibody: Application statement in manufacturer's website as follows: FC-Quality tested. https://www.biolegend.com/en-us/products/brilliant-violet-605-anti-mouse-human-cd45r-b220-antibody-7870?Clone=RA3-6B2&pdf=true&displayInline=true&leftRightMargin=15&topBottomMargin=15&filename=Brilliant%20Violet%20605â„¢%20anti-mouse/human%20CD45R/B220%20Antibody.pdf

2. CD16/32 Block antibody: Application statement in manufacturer's website as following: the antibody is validated for use in Flow cytometry, Blocking, IH-frozen, IP. https://www.bdbiosciences.com/content/bdb/paths/generate-tds-document.cn.553142.pdf

3. CD115-BV421 antibody: Application statement in manufacturer's website as following: FC-Quality tested. https://www.biolegend.com/en-us/products/brilliant-violet-421-anti-mouse-cd115-csf-1r-antibody-8971?pdf=true&displayInline=true&leftRightMargin=15&topBottomMargin=15&filename=Brilliant%20Violet%20421â„¢%20anti-mouse%20CD115%20(CSF-1R)%20Antibody.pdf

4. CD117-BV650 antibody: Application statement in manufacturer's website as following: FC-Quality tested. https://www.biolegend.com/en-us/products/brilliant-violet-650-anti-mouse-cd117-c-kit-antibody-19797?Clone=2B8&pdf=true&displayInline=true&leftRightMargin=15&topBottomMargin=15&filename=Brilliant%20Violet%20650â„¢%20anti-mouse%20CD117%20(c-kit)%20Antibody.pdf

5. CD11b-Percp-Cy5.5 antibody: Application statement in manufacturer's website as following: the antibody is validated for use in Flow cytometry. https://www.bdbiosciences.com/content/bdb/paths/generate-tds-document.us.550993.pdf

6. CD11b- FITC antibody: Application statement in manufacturer's website as following: the antibody is validated for use in Flow cytometry and Immunofluorescence. https://www.bdbiosciences.com/content/bdb/paths/generate-tds-document.us.557396.pdf

7. CD11c- PE antibody: Application statement in manufacturer's website as following: the antibody is validated for use in Flow cytometry, IHC, IHC(F), ICC/IF, ELISA, FN. https://www.thermofisher.cn/order/genome-database/dataSheetPdf?producttype=antibody&productsubtype=antibody_primary&productId=12-0114-82&version=261

8. CD24-BV510 antibody: Application statement in manufacturer's website as following: FC-Quality tested. https://d1spbj2x7qk4bg.cloudfront.net/en-us/products/brilliant-violet-510-anti-mouse-cd24-antibody-9925?pdf=true&displayInline=true&leftRightMargin=15&topBottomMargin=15&filename=Brilliant%20Violet%20510™%20anti-mouse%20CD24%20Antibody.pdf&v=20220831123135

9. CD45-CoraLite®488 antibody: Application statement in manufacturer's website as following: FC tested. https://www.ptglab.com/products/pictures/pdf/CL488-65121.pdf

10. CD45-APC/Cy7 antibody: Application statement in manufacturer's website as following: FC-Quality tested. https://d1spbj2x7qk4bg.cloudfront.net/en-us/products/apc-cyanine7-anti-mouse-cd45-antibody-2530?pdf=true&displayInline=true&leftRightMargin=15&topBottomMargin=15&filename=APC/Cyanine7%20anti-mouse%20CD45%20Antibody.pdf&v=20220831123135

11. CD45-BV510 antibody: Application statement in manufacturer's website as following: FC-Quality tested. https://d1spbj2x7qk4bg.cloudfront.net/en-us/products/brilliant-violet-510-anti-mouse-cd45-antibody-7995?pdf=true&displayInline=true&leftRightMargin=15&topBottomMargin=15&filename=Brilliant%20Violet%20510™%20anti-mouse%20CD45%20Antibody.pdf&v=20220831123135

12. CD64-BV421 antibody: Application statement in manufacturer's website as follows: FC-Quality tested. https://d1spbj2x7qk4bg.cloudfront.net/en-us/products/brilliant-violet-421-anti-mouse-cd64-fcgammari-antibody-8992?pdf=true&displayInline=true&leftRightMargin=15&topBottomMargin=15&filename=Brilliant%20Violet%20421™%20anti-mouse%20CD64%20(FcγRI)%20Antibody.pdf&v=20220831123135

13. Clec5a-Alexa Fluor 488 antibody: Application statement in manufacturer's website as follows: Flow Cytometry tested. https://resources.rndsystems.com/pdfs/datasheets/fab1639g.pdf?v=20221106&_ga=2.107594306.1349784613.1667731546-1574924739.1667731546

14. Cx3cr1-APC-Fire750 antibody: Application statement in manufacturer's website as follows: FC-Quality tested. https://d1spbj2x7qk4bg.cloudfront.net/en-us/products/apc-fire-750-anti-mouse-cx3cr1-antibody-13846?pdf=true&displayInline=true&leftRightMargin=15&topBottomMargin=15&filename=APC/Fire™%20750%20anti-mouse%20CX3CR1%20Antibody.pdf&v=20220217070239

15. F4/80-BV421 antibody: Application statement in manufacturer's website as follows: FC-Quality tested, IHC-F-Verified, SB-Reported in the literature, not verified in house. https://d1spbj2x7qk4bg.cloudfront.net/en-us/products/brilliant-violet-421-anti-mouse-f4-80-antibody-7199?pdf=true&displayInline=true&leftRightMargin=15&topBottomMargin=15&filename=Brilliant%20Violet%20421™%20anti-mouse%20F4/80%20Antibody.pdf&v=20220831123135

16. F4/80-PE antibody: Application statement in manufacturer's website as follows: Flow cytometry. https://www.bdbiosciences.com/content/bdb/paths/generate-tds-document.us.565410.pdf

17. Ly6c-PE-Cy7 antibody: Application statement in manufacturer's website as follows: Flow cytometry. https://www.thermofisher.cn/order/genome-database/dataSheetPdf?producttype=antibody&productsubtype=antibody_primary&productId=25-5932-82&version=261

18. Ly6c-FITC antibody: Application statement in manufacturer's website as follows: FC-Quality tested. https://d1spbj2x7qk4bg.cloudfront.net/en-us/products/fitc-anti-mouse-ly-6c-antibody-4896?pdf=true&displayInline=true&leftRightMargin=15&topBottomMargin=15&filename=FITC%20anti-mouse%20Ly-6C%20Antibody.pdf&v=20220831123135

19. Ly6g-APC antibody: Application statement in manufacturer's website as follows: Flow cytometry. https://www.bdbiosciences.com/content/bdb/paths/generate-tds-document.us.560599.pdf

20. Ly6g-FITC antibody: Application statement in manufacturer's website as follows: Flow cytometry, IHC, IHC(F), ICC/IF https://www.thermofisher.cn/order/genome-database/dataSheetPdf?producttype=antibody&productsubtype=antibody_primary&productId=11-9668-82&version=261

21. Marco-FITC antibody: Application statement in manufacturer's website as follows: Flow cytometry. https://resources.rndsystems.com/pdfs/datasheets/fab2956f.pdf?v=20221106&_ga=2.18039033.1349784613.1667731546-1574924739.1667731546

22. MHCII-PE antibody: Application statement in manufacturer's website as follows: IHC, Flow, Neu, FN, BLOCK, IV. https://www.thermofisher.cn/order/genome-database/dataSheetPdf?producttype=antibody&productsubtype=antibody_primary&productId=12-5321-82&version=261

23. MHCII-FITC antibody: Application statement in manufacturer's website as follows: FC-Quality tested. https://d1spbj2x7qk4bg.cloudfront.net/en-us/products/fitc-anti-mouse-i-a-i-e-antibody-366?pdf=true&displayInline=true&leftRightMargin=15&topBottomMargin=15&filename=FITC%20anti-mouse%20I-A/I-E%20Antibody.pdf&v=20220421115313

24. Muc1-FITC antibody: Application statement in manufacturer's website as follows: ELISA, Flow Cytometry, Immunocytochemistry/Immunofluorescence, Immunohistochemistry, Immunohistochemistry-Paraffin. https://d1spbj2x7qk4bg.cloudfront.net/en-us/products/fitc-anti-mouse-i-a-i-e-antibody-366?pdf=true&displayInline=true&leftRightMargin=15&topBottomMargin=15&filename=FITC%20anti-mouse%20I-A/I-E%20Antibody.pdf&v=20220421115313

25. Pdl1-FITC antibody: Application statement in manufacturer's website as follows: Flow Cytometry. https://www.novusbio.com/PDFs5/FAB9078F.pdf

26. Sifglec-F-Percp-eFluor 710 antibody: Application statement in manufacturer's website as follows: Flow Cytometry. https://www.thermofisher.cn/order/genome-database/dataSheetPdf?producttype=antibody&productsubtype=antibody_primary&productId=46-1702-82&version=261

27. Goat anti-rabbit IgG-HRP: Application statement in manufacturer's website as follows: Suitable for IHC-P, WB, ELISA, Immunomicroscopy, Dot blot, ICC, IHC-Fr. https://www.abcam.com/Goat-Rabbit-IgG-HL-HRP-ab6721.html

28. goat anti-mouse IgG-HRP: Application statement in manufacturer's website as follows: Suitable for IHC-P, WB, ELISA, Immunomicroscopy, Dot blot, ICC, IHC-Fr. https://www.abcam.com/goat-mouse-igg-hl-hrp-ab6789.html

29. Il1β antibody: Application statement in manufacturer's website as follows: Flow Cytometry, IF, WB. https://www.cellsignal.cn/datasheet.jsp?productId=12703&images=1&size=A4

30. Il18 antibody: Application statement in manufacturer's website as follows: WB. https://www.cellsignal.cn/datasheet.jsp?productId=57058&images=1&size=A4

31. Nek7 antibody: Application statement in manufacturer's website as follows: WB. https://www.cellsignal.cn/datasheet.jsp?productId=3057&images=1&size=A4

32. Nlrp3 antibody: Application statement in manufacturer's website as follows: WB, IP https://www.cellsignal.cn/datasheet.jsp?productId=15101&images=1&size=A4

33. Pro-caspase antibody: Application statement in manufacturer's website as follows: WB, IP https://www.cellsignal.cn/datasheet.jsp?productId=2225&images=1&size=A4

34. β-actin antibody: Application statement in manufacturer's website as follows: WB, IH, IF, FC https://www.cellsignal.cn/datasheet.jsp?productId=3700&images=1&size=A4

35. anti-Il18 antibody: InVivo IL-18 neutralization, https://d2a7cdyquyl45u.cloudfront.net/tds-sheets/BE0237-tds.pdf

Eukaryotic cell lines

Policy information about cell lines and Sex and Gender in Research

Cell line source(s)	RAW264.7 (Cat# TIB-71 from ATCC)

Authentication	Cell lines were authenticated using STR analysis as described in 2012 in ANSI Standard (ASN-0022)
Mycoplasma contamination	The cell lines tested negative for mycoplasma contamination
Commonly misidentified lines (See ICLAC register)	None

Animals and other research organisms

Policy information about studies involving animals; ARRIVE guidelines recommended for reporting animal research, and Sex and Gender in Research

Laboratory animals	Normal male C57BL/6N mice purchased from Shanghai Slac Laboratory Animal Co., Ltd., and Il18-/- mice provided kindly by Professor Rongbin Zhou, were kept in SPF conditions at the College of Pharmacy, Zhangjiang Campus, Fudan University. Eight- to twelve-week-old (20-25 g) mice were subjected to tissue collection or macrophage isolation. The euthanasia of animals was performed by carbon dioxide (CO2) inhalation. The permission for animal experiments was granted by the Research Ethics Committees of Zhongshan Hospital.
Wild animals	The study did not involve wild animals
Reporting on sex	Only male mice were applied in the study.
Field-collected samples	The study did not involve field-collected samples
Ethics oversight	The permission for animal experiments was granted by the Research Ethics Committees of Zhongshan Hospital.

Note that full information on the approval of the study protocol must also be provided in the manuscript.

Flow Cytometry

Plots

Confirm that:

- [x] The axis labels state the marker and fluorochrome used (e.g. CD4-FITC).
- [x] The axis scales are clearly visible. Include numbers along axes only for bottom left plot of group (a 'group' is an analysis of identical markers).
- [x] All plots are contour plots with outliers or pseudocolor plots.
- [x] A numerical value for number of cells or percentage (with statistics) is provided.

Methodology

Sample preparation	Macrophages were purified following previously published protocols. Briefly, microglia were isolated from brains by subjecting cell suspension to density gradient centrifugation (with a 0%, 30%, 37%, and 70% Percoll density gradient) for 40 min at 200 g after HBSS perfusion and enzymatic digestion in HBSS containing collagenase Ⅳ (Sigma, 0.5 mg/ml), DNase Ⅰ (Worthington, 0.02 mg/ml), dispase (Sigma, 2 mg/ml) and Nα-tosyl-L-lysine chloromethylketone hydrochloride (TLCK, Sigma, 0.1 mg/ml) for 20 min at room temperature63, 64. Kupffer cells and liver-recruited macrophages were extracted from livers by a two-step perfusion digestion in situ of the hepatic portal vein with collagenase Ⅳ (Sigma, 0.5 mg/ml) and DNase Ⅰ (Worthington, 0.01 mg/ml) and then separated from each other by 0%, 11.2%, 17.6 and 24% OptiprepTM density gradient centrifugation for 18 min at 1400 g65. Lung-resident and recruited macrophages were purified from the lung after first carrying out a PBS perfusion and enzymatic digestion of lung tissue by collagenase Ⅳ (Sigma, 0.5 mg/ml) and DNase Ⅰ (Worthington, 0.02 mg/ml) for 45 min at 37 ℃66, 67. Spleen-resident and recruited macrophages were extracted after carrying out tissue grinding and RBC lysis for 3 min. Intestinal macrophages were extracted through enzymatic digestion of small or large intestine tissues in HBSS with collagenase Ⅳ or VIII (Sigma, 0.75 mg/ml) and DNase Ⅰ (Worthington, 0.04 mg/ml) for 10 min at 37 ℃ after carrying out an epithelial segregation in HBSS with EDTA and FBS for 40 min at 37 ℃68. Peritoneal macrophages were isolated by carrying out peritoneal lavage with PBS. BMDMs were obtained from bone marrow cells that were treated within RBC lysis in DMEM with 10% FBS and mouse M-CSF.<br><br>Prepared cell suspensions from mouse tissues were incubated with fluorescently labeled antibodies (1:200 dilution in 2%FCS in PBS) directed against cell surface markers. Cell surface labeling was performed on ice for 30 min.
Instrument	Cells were iltered through a 40 mm cell strainer (BD Falcon, 352340), and analyzed on Beckman Counter cytoFLEX LX, or sorted by BD influx or BD FACS AriaIII with 100-µm nozzle.
Software	flow cytometry (FCM) data were analyzed by FlowJoTM 10.
Cell population abundance	Macrophages purity were identified by FACS test of post-sort fraction,and the average purity of macrophage populations was greater than 98%

Gating strategy

Cell debris were removed by FSC/SSC gating, then the sticky cells were excluded by trigger pulse width gating.

- [x] Tick this box to confirm that a figure exemplifying the gating strategy is provided in the Supplementary Information.
